# Psychomotor abilities of candidates for Polish Special Forces

**DOI:** 10.1038/s41598-022-09138-4

**Published:** 2022-03-24

**Authors:** Wojciech Paśko, Przemysław Guła, Maciej Brożyna, Bartosz Dziadek, Emilian Zadarko, Maciej Śliż, Klementyna Polak, Krzysztof Przednowek

**Affiliations:** 1 Institute of Physical Culture Sciences, Medical College of Rzeszów University, Rzeszów University, Rzeszow, Poland; 2 Institute of Medical Sciences, Medical College of Rzeszów University, Rzeszów University, Rzeszow, Poland

**Keywords:** Occupational health, Quality of life, Human behaviour, Visual system

## Abstract

Combat tasks involving special units often put a mental and physical strain on the soldiers. During the military operation, soldiers may struggle with multiple difficulties such as extreme physical effort, sleep deprivation, weather conditions, limited access to food and stress. These circumstances require a high level of cognitive ability (including psychomotor abilities) to overcome the physiological stress response and to be able to quickly and efficiently make decisions under stressful situations, especially in combat. The aim of the study was to assess the level of the psychomotor abilities of candidates for the Polish Special Forces. The study comprised 48 candidates for the Polish Special Forces (age: $30.06 \pm 2.87$), 40 athletes (age: $27.93 \pm 3.91$) and 40 non-athletes (age: 24). The study was performed using Test2Drive software. Four computer-based tests were used to assess the following psychomotor abilities: simple reaction time, choice reaction time, hand-eye coordination and two-dimensional visuomotor coordination/spatial orientation test (SPANT). The analysis demonstrated statistically significant differences in reaction time and motor time between the study groups. The shortest reaction time in each test was observed in athletes, while the shortest motor time was observed in soldiers. All the psychomotor tests, except for the number of correct answers in SPANT, demonstrated statistically significant differences between the studied groups. It was also found that military training had a positive effect on the motor time in every psychomotor test. As regards the reaction time, it was observed that the athletes were characterised with better reaction times than the special forces candidates. The study has confirmed that military training and sports training have a positive effect on the level of psychomotor abilities, especially motor time.

## Introduction

Combat tasks involving special units often put a mental and physical strain on the soldiers. During a military operation, soldiers may encounter difficult weather conditions, limited access to food, logistical changes or difficult infiltration and exfiltration circumstances^1^. A mission may last from several days to several weeks in politically varied areas, where soldiers may even be immobilized for up to 12 days^2^. Special forces can expect to participate in combat at any time, which is associated with their constant readiness for external stimuli^3–5^. The special unit training and selection process is characterized by extreme physical effort lasting from 16 to 22 hours a day and sleep deprivation. The soldiers are exposed to strenuous physical exercise, such as long loaded marches/runs involving distances of about 90 km with loads of up to 40% of their body weight^6^. These activities require a high level of cognitive ability to overcome the physiological response to stress^7^. Therefore, a candidate for a soldier must demonstrate appropriate psychophysical skills^8^.

The psychomotor abilities are crucial for the soldiers to be able to quickly and efficiently make effective decisions under stressful situations. Many studies on psychomotor abilities in soldiers have been performed after survival training, during which they were subjected to extreme effort and sleep deprivation^9–13^. When changes in motor coordination and reaction speed were assessed in Polish Air Force cadets after 36 hours of survival training, during which the participants were deprived of sleep, no significant differences in these parameters were observed^9^. Polish special unit soldiers participated in similar studies to assess the impact on their psychomotor abilities^10^.The impact of survival training on psychomotor abilities was also assessed in Polish military pilots^12^. Other studies analysed alterations in psychomotor abilities for each day of survival training^11,14^. Changes in reaction time were also analysed in combat situations, where soldiers were exposed to sleeplessness and anxiety^15,16^.

Other studies assessed the effect of caffeine on the improvement of cognitive functions and marksmanship effectiveness in soldiers^13,17,18^. Whereas, DeVocht et al.^19^ studied the impact of a chiropractic manipulation session on the reaction speed in special unit soldiers. The impact of high temperature on the level of cognitive abilities in soldiers and the importance of acclimatization in specific weather environments have also been investigated^20^. Shukitt-Hale et al.^21,29^ assessed the impact of a low-calorie diet in physically active soldiers on their reaction speed. In a study carried out by Flegr et al.^22^, the impact of the presence of the D antigen and age on the psychomotor abilities was evaluated in Czech soldiers. The analysis of reaction time has also been used for the preliminary diagnosis of concussion in soldiers on the battlefield^23,24^.

Studies comparing the level of psychomotor abilities in elite soldiers versus control groups have also been conducted^5,25,26^. A comparative study of reaction speed under stressful situations involving a group of elite and non-elite Spanish soldiers was carried out by Sanchez et al.^25^. They assessed changes in the reaction times of Spanish soldiers due to unexpected stimuli in the form of an attack on a base or checkpoint. In another work, Meško et al.^26^ compared the level of psychomotor abilities in Slovenian military pilots versus a control group.

The literature review indicated the significant role played by psychomotor abilities in soldiers while performing their tasks, especially in combat.Psychomotor skills can determine the success of the task performed by quick and correct responding to the situation. Therefore, the aim of our study was to assess the psychomotor abilities in candidates for the elite unit of the Polish Special Forces.The aim of the study was also demonstrating whether military training significantly influences on the level of psychomotor skills. In order to thoroughly analyse and understand the characteristic features of this elite group of soldiers, their performance was compared to a group of athletes and a group of non-athletes (control group).The results obtained will allow to determine which psychomotor skills should be characterized a candidate for special forces to best meet the demands will encounter during combat tasks as a special force operator.

## Material

The study comprised 48 candidates for the Polish Special Forces (age: $30.06 \pm 2.87$ years), compared to a group of 40 athletes (age: $27.93 \pm 3.91$ years) and a group of 40 non-athletes (control group) (age: 24 years).In the study took part all candidates for the Polish Special Forceswho have completed the first (fitness tests) and the second (mountain survival camp)of the selection stages. The group of athletes consisted of 20 football players (1st division), 10 volleyball players (2nd division) and 20 handball players (1st division).All the athletes participated in studies are playing in professional teams from the Subcarpathian region. The number of respondents were determined by the number of professional team consents. The control group consisted of students from the University of Rzeszowof the same age. All students were studying at the last year of Master’s degree studies, none of them were professional player. Each participant expressed their consent to participate in the study.Written informed consent was obtained from all the participants. In the study participated only adult men who were rested and who do not have health problem. None of the participants was treated pharmacologically and was not injured during the studies.

The study scope and design were approved by the Ethics Committee of the University of Rzeszów/Poland (resolution 3/01/2021). All procedures performed in studies involving human participants were in accordance with the ethical standards of the Ethics Committee of the University of Rzeszów and with the 1964 Helsinki Declaration and its later amendments.

## Methods

The study assessed the psychomotor abilities of candidates for the Polish Special Forces versus a group of athletes and a group of non-athletes.The study followed the STROBE checklist^27^.

**Figure 1:**
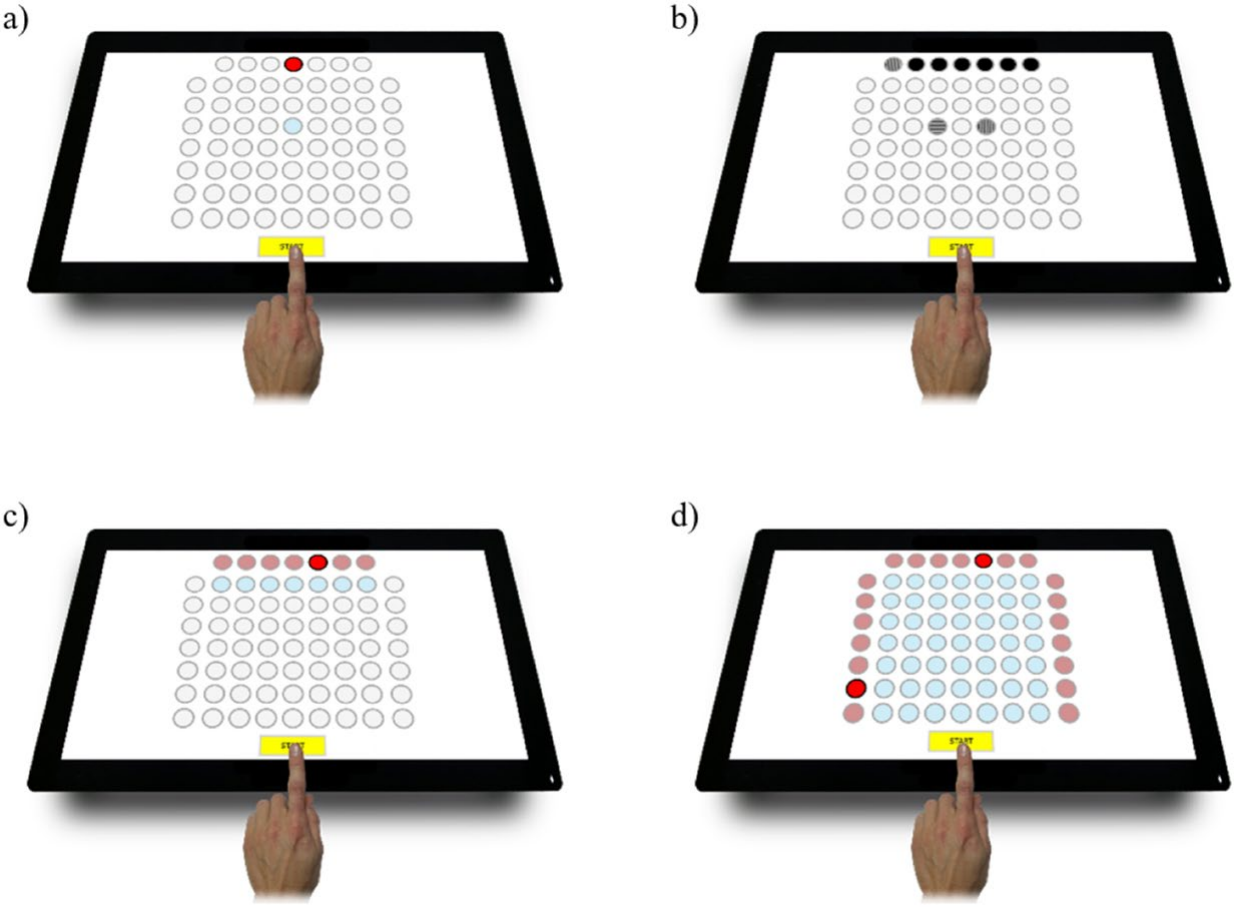
Reaction panel of the Test2Drive system; (**a**) SIRT, (**b**) CHORT, (**c**) HECOR, (**d**) SPANT^29^.

The Test2Drive computer system was used to assess the level of psychomotor abilities^28^. Four tests were used in the study to measure, for example, simple reaction time (SIRT), choice reaction time (CHORT), hand-eye coordination (HECOR) and two dimensional visuomotor coordination (spatial anticipation test—SPANT)(Fig. 1). Each participant has performed the tests in the following order:

- Test SIRT—The measurement is used to evaluate the simple reaction time. The participant’s task was to move finger from the “START” field to the blue point as quick as it is possible after appearing the visual stimulus, which was red point. During the test, points did not change their position.
- Test CHORT—The measurement is used to evaluate the response time with a choice. The participant’s task was to respond appropriately to stimuli in the form of vertical, horizontal and diagonal lines. During the appearance of diagonal lines, the participant’s task was to remain unresponsive and the finger should remain on the “START” field, while in the case of vertical and horizontal lines, it was to react to the appropriate stimulus with the correct choice. Incorrect responses were also measured during the test.
- Test HECOR—The measurement was used to assess hand-eye coordination. The participant’s task was to move finger from the “START” field to the appropriate blue field, which was placed under the illuminated red field.
- Test SPANT—The measurement was used to assess hand-eye coordination with the use of complex spatial information. The participant’s task was to select the appropriate blue field, which was the intersection of two blue points on the left and top of the screen. The measurement started from the “START” field.

Each of the tests involved the measurement of the reaction time (RT) and motor time (MT).Reaction time (RT) was measured from the appearance of the visual stimulus until finger was removed from the “START” field. The motor time (MT) was measured from the moment when finger was moved from the “START” field to the answer selection. In each type of test, the stimuli appeared at different time intervals. The study subjects performed tasks in a room facilitating concentration, in a standing position, using the index finger of the dominant hand.The temperature during the studies was 19$^\circ$C. Each study test was preceded by a trial test that gave the subject a chance to become acquainted with the test procedure. The full description of the selected tests and the method of conducting the study have been described in previous publications^29,30^.

Descriptive characteristics (number, arithmetic mean, standard deviation) were used in the analyses aimed at assessing the selected psychomotor abilities in the study groups. In addition, the normal distribution of variables was verified (Shapiro-Wilk test) and the statistical significance of differences between the levels of reaction time and motor time obtained in individual groups were determined (Kruskal–Wallis test, Wilcoxon test).The effect size for Kruskal–Wallis test was calculated using formula^31^:

1 $$\begin{aligned} \eta _{\text {H}}^{2}=\dfrac{H-k+1}{n-k} \end{aligned}$$

where: *H*—the value obtained in the Kruskal–Wallis test (the Kruskal–Wallis H-test statistic), $\eta _{\text {H}}^{2}$—eta-squared estimate assumes values from 0 to 1 and multiplied by 100% indicates the percentage of variance in the dependent variable explained by the independent variable, *k*—the number of groups, *n*—the total number of observations. The effect size for Wilcoxon test was calculated using formula^31^:

2 $$\begin{aligned} r=\dfrac{Z}{\sqrt{N}} \end{aligned}$$

where: *Z*—standardized value for the U-value, *r*—correlation coefficient where *r* assumes the value ranging from −1.00 to 1.00, *N*—the total number of observations on which Z is based. All calculations and analyses were performed using the GNU R environmentand at the level of significance $\alpha$ = 0.05^32^

### Ethics declarations

The study was conducted in accordance with the Declaration of Helsinki, and the protocol was approved by the Ethics Committee of the University of Rzeszow / Poland (resolution 10/02/2020). Before the study, the participants gave their informed consent to participate in the study.

## Results

Table 1 shows the averaged results of the psychomotor tests for each group. The shortest reaction time (RT) in each test was observed in athletes, while the shortest motor time (MT) was observed in soldiers. The group of soldiers demonstrated the longest reaction time in SIRT (350.6 ms) and HECOR (421 ms). The control group had the longest motor time in the individual tests. The soldiers obtained the highest number of correct answers in CHORT and SPANT. The athletes obtained the lowest number of correct answers (93.3%). The same median of correct answers in SPANT was observed in all groups (95%). All test results were statistically significant with the exception of the correct answers in SPANT.

**Table 1:** Numeral characteristics of psychomotor abilities of groups.

Variable	Soldiers		Athlete		Non-athlete		*p*	$\eta _{\textit{H}}^{2}$
	$\bar{x} \pm sd$	*Me*	$\bar{x} \pm sd$	*Me*	$\bar{x} \pm sd$	*Me*		
**SIRT**								
RT [ms]	$350.6 \pm 35.2$	348.5	$333.0 \pm 34.0$	331.5	$336.4 \pm 49.8$	323	0.0285*	0.03
MT [ms]	$165.2 \pm 38.9$	165.0	$174.3 \pm 41.6$	177.5	$282.2 \pm 71.8$	270.5	0.0001***	0.45
**CHORT**								
RT [ms]	$695.0 \pm 38.8$	687.0	$645.6 \pm 76.8$	633.5	$711.7 \pm 63.6$	701.5	0.0002***	0.14
MT [ms]	$186.3 \pm 42.3$	182.0	$209.2 \pm 72.9$	189	$320.3 \pm 93.9$	300.5	0.0001***	0.41
c.r. [%]	$96.7 \pm 5.1$	100	$93.3 \pm 6.0$	92	$94.8 \pm 8.3$	96	0.0114*	0.04
**HECOR**								
RT [ms]	$421 \pm 32.3$	417	$392.5 \pm 36.0$	390.5	$413.0 \pm 40.9$	406	0.0006***	0.09
MT [ms]	$210.3 \pm 44.6$	205.5	$221.1 \pm 44.5$	217.5	$349.6 \pm 116.1$	352	0.0001***	0.43
**SPANT**								
RT [ms]	$631.2 \pm 87.3$	622	$584.4 \pm 79.3$	578	$636.8 \pm 112.6$	648	0.0237*	0.03
MT [ms]	$208.8 \pm 46.3$	199.5	$248.9 \pm 75.6$	233.5	$375.0 \pm 110.6$	362.5	0.0001***	0.43
c.r. [%]	$93.5 \pm 8.4$	95	$93.4 \pm 6.4$	95	$89.8 \pm 15.4$	95	0.7214	− 0.03

*SIRT* simple reaction time, *CHORT* choice reaction time, *HECOR* hand-eye coordination test, *SPANT* spatial anticipation test, *RT* reaction time, *MT* movement time, *c.r.* correct responses, $$*>0.05, **>0.01,***>0.001$$,$$\eta _{\text {H}}^{2}$$—effect size for the Kruskal–Wallis test.

The differences in reaction time and motor time between the groups are presented in Table 2. In SIRT, the greatest differences between the soldiers and the control group were observed in motor time, with the time obtained by soldiers being 117 ms shorter. Regarding the motor time, similar correlations occurred in each test, with the soldiers achieving the shortest time compared to other groups. In the case of the reaction time in SIRT, the fastest responders were athletes, whose average time was 3.4 ms quicker than the control group and 17.6 ms quicker than the soldiers. In CHORT and SPANT, the greatest differences in reaction time occurred between the athletes and the control group, with the athletes having 66.1 ms and 52.4 ms quicker reaction times, respectively. In CHORT, the reaction time of soldiers did not differ significantly from the average time obtained in the control (untrained) group; however, the soldiers’ reaction was 16.7 ms quicker. In HECOR, the greatest difference in RT was found between soldiers and athletes, with athletes obtaining a 28.5 ms quicker average time. The difference between the athletes and the control group in HECOR (8 ms) was not statistically significant. In the assessment of the level of correct responses obtained in CHORT and SPANT between the soldiers and the other groups, the greatest differences were found versus the athletes (CHORT) and the control group (SPANT). The most similar results in terms of the correct answers were obtained in the group of soldiers and athletes, amounting to 0.1 percent.

**Table 2:** Differences between groups.

Variable	S versus A			S versus N-A			A versus N-A		
	*d*	*p*	*r*	*d*	*p*	*r*	*d*	*p*	*r*
**SIRT**									
RT [ms]	17.6	0.0275*	0.23	14.2	0.0214*	0.25	− 3.4	0.6755	0.05
MT [ms]	− 9.1	0.2777	− 0.12	− 117	0.0001*	− 0.76	− 107.9	0.0001*	− 0.7
**CHORT**									
RT [ms]	49.4	0.0003*	0.39	− 16.7	0.1924	− 0.14	− 66.1	0.0001*	− 0.47
MT [ms]	− 22.9	0.1785	− 0.14	− 134	0.0001*	− 0.76	− 111.1	0.0001*	− 0.61
c.r. [%]	3.4	0.0034*	0.3	1.9	0.4498	0.07	− 1.5	0.0475*	− 0.21
**HECOR**									
RT [ms]	28.5	0.0002*	0.39	8	0.1179	0.17	20.5	0.0198*	− 0.26
MT [ms]	− 10.8	0.2164	− 0.13	− 139.3	0.0001*	− 0.74	− 128.5	0.0001*	− 0.69
**SPANT**									
RT [ms]	46.8	0.0073*	0.29	− 5.6	0.9933	0.00	− 52.4	0.0433*	− 0.23
MT [ms]	− 40.1	0.0128*	− 0.27	− 166.2	0.0001*	− 0.76	− 126.1	0.0001*	− 0.59
c.r. [%]	0.1	0.4285	0.08	3.7	0.6943	0.04	3.6	0.6845	− 0.04

*SIRT* simple reaction time, *CHORT* choice reaction time, *HECOR* hand-eye coordination test, *SPANT* spatial anticipation test, *RT* reaction time, *MT* movement time, *c.r.* correct responses, *d* difference, *p* statistical probability, *r* effect size for the the Mann–Whitney–Wilcoxon test.

*Statistical significance.

**Figure 2:**
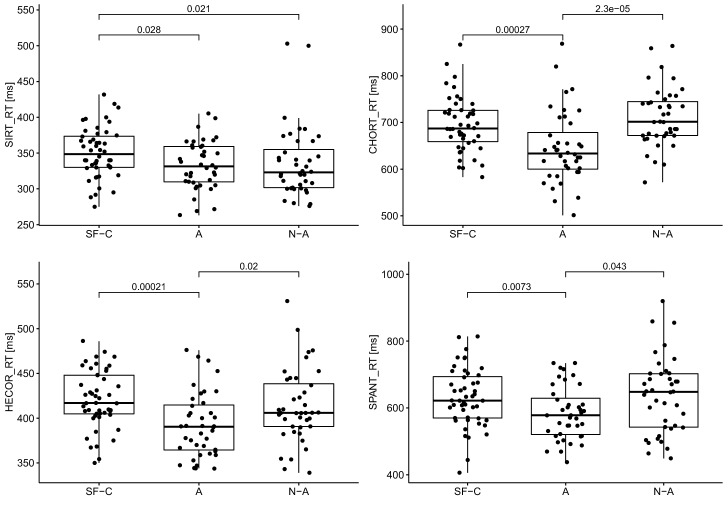
Reaction time for each groups.

The results of the simple reaction time assessment in individual tests, including the statistical significance of the differences between the analysed groups, are presented in Fig. 2. Statistical significance in SIRT was obtained in the comparisons between the soldiers and both the athlete and control groups. Regarding the reaction time in CHORT, statistical significance was obtained in the comparisons between the athletes and both the soldier and control groups. In HECOR, statistical significance was also obtained in the comparisons between the athletes and both the soldier and control groups. Similarly, in the case of the reaction time in SPANT, statistical significance was obtained in the comparisons between the athletes and both the soldier and control groups.

**Figure 3:**
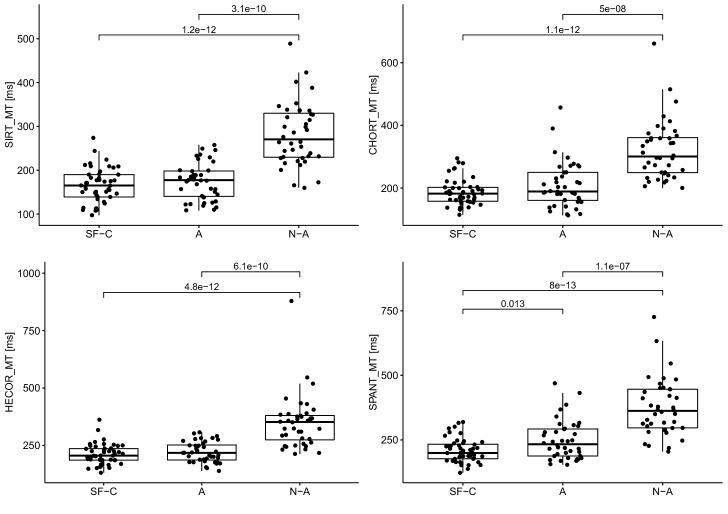
Motor time for each groups.

When assessing the statistical significance of the motor time differences between the respective groups (Fig. 3) in SIRT, CHORT and HECOR, statistical significance was obtained in the comparisons between the controls and both the soldier and the athlete groups. However, in SPANT, statistically significant differences were observed between all groups.

**Figure 4:**
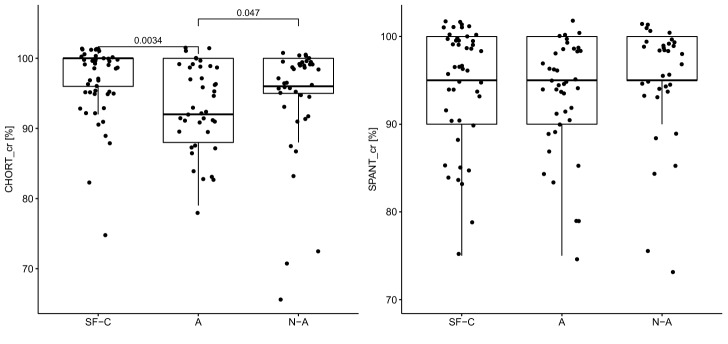
Choosing the correct answers for each group.

Figure 4 shows the results and statistical significance between the correct answers in CHORT and SPANT. Statistical significance was observed only in CHORT in the comparisons between the athletes and both the soldier and the control groups. However, in SPANT, there were no statistically significant differences in the number of correct answers.

## Discussion

The main objective of the study was to assess the level of psychomotor abilities of candidates for the elite unit of the Polish Special Forces. The study has shown that military training is associated with motor time improvement. The greatest differences in motor time, visible in each psychomotor test, occurred between the group of soldiers and the group of non-athletes. However, the reaction time differences between the groups were small. In case of choosing the correct answers, only CHORT demonstrated significant differences between the selected groups. The results show that there is a relationship between military training and psychomotor abilities. Johnston and Catano^33^ also suggest that the type of tasks performed at work affects cognitive abilities. A study by Hickey et al.^34^ has confirmed that psychomotor abilities improve through military training. Similar conclusions were reached by Różański et al.^10^, with an improvement in psychomotor abilities for special force soldiers after survival training. Whereas, Marx et al.^35^ observed that, while staying in the war zone in Iraq, soldiers improved their reaction speeds in comparison to before their deployment. It was also observed that high temperature had a negative effect on psychomotor test results, while 10-day acclimatization in the local weather conditions had a positive effect on the test results^20^. The analysis carried out by Forgues^36^ demonstrated that candidates for military pilots who scored well in psychomotor tests were successful in the pilot selection process.

In most psychomotor tests, the best reaction time was achieved by the group of athletes; however, the differences with the group of candidates for special units were small. In the case of simple reaction time, the control group obtained the shortest reaction time in terms of the median value. The results obtained in each test suggest that reaction time is determined by individual characteristics. A study by Xiaoli et al.^37^, who assessed the long-term influence of the environment and the tasks performed by soldiers, demonstrated no significant differences in reaction time. On the other hand, Tomczak et al.^9^ observed no change in reaction time after 36 hours of survival training. Changes in the level of psychomotor abilities after survival training were assessed in Polish military pilots, and only a slight change in motor skills and divisibility of attention was found^12^.

Candidates for the elite Polish Special Forces unit had the best motor time in any psychomotor test. The analysis showed that statistically significant differences occurred in each test between the candidate group and the group of non-athletes, as well as between the group of athletes and the group of non-athletes. In the two-dimensional visuomotor coordination test, the differences in motor time were statistically significant between all groups. The results suggest that sports training and specialized military training significantly improve motor time. A study including a group of elite and non-elite soldiers of the Spanish army showed that the elite group soldiers reacted faster in stressful situations^25^. In another study by Sánchez-Molina et al.^5^ it was also concluded that elite soldiers respond better in combat simulation. Tornero et al.^38^ showed that elite soldiers react better in a situation involving combat stress. Comparisons of Slovenian military pilots with a control group demonstrated a higher level of psychomotor abilities in the military pilots^26^.

The analysis of the correctness of the answers in the reaction time test combined with choice showed that the group of candidates for the elite unit of the Polish Special Forces was characterized by the highest level of error-free reactions. There are statistically significant differences between the group of soldiers and athletes, and between the group of athletes and non-athletes. Candidates for special units obtained the mean value of 96.7 %for correct answers. In the case of the two-dimensional visuomotor coordination test, no statistically significant differences were observed. In their work, Radakovic et al.^20^ found a reduced number of correct answers during psychomotor tests due to a lack of acclimatization of soldiers functioning in difficult conditions. Lisowski and Mihuta^39^ emphasized the key importance of psychomotor abilities in the effective performance of combat tasks by soldiers. One study demonstrated the potential of using cognitive training to improve marksmanship. Correct response training reduced the number of simulated civilian casualties^40^.

The limitations of the study include the lack of comparison of the candidates for the Polish Special Forces with the current elite unit operators. A group consisting of current special operators would help to determine how psychomotor abilities change during training and operation as a special unit soldier. Many studies show how acclimatization and military training affect psychomotor abilities^8–13,15,16,20^. In addition, the study was conducted in laboratory conditions, while special forces soldiers often work in stressful situations that can significantly reduce the level of psychomotor abilities^5,25,35,38^. This is why it is also worth assessing the psychomotor abilities by means of tests performed under conditions similar to combat operations.

## Conclusion

The analysis of the data led to the following conclusions:

1. All the psychomotor tests, except for the number of correct answers in SPANT, demonstrated statistically significant differences between the studied groups.
2. In the case of reaction time, the differences between the groups were small, which means thatCandidates for the Polish Special Forces has little influence on its improvement. Reaction time may be influenced by individual characteristics.
3. Candidates for the Polish Special Forces had the best motor time in any psychomotor test. The largest differences were observed between the soldiers and non-athletes, as well as between the athletes and non-athletes. This means that sports training and military training significantly improve motor time.

## Data Availability

The datasets generated and analysed during the current study are available from the corresponding author on reasonable request. All data generated or analysed during this study are included in this published article.
